# In vivo tau pathology is associated with synaptic loss and altered synaptic function

**DOI:** 10.1186/s13195-021-00772-0

**Published:** 2021-02-05

**Authors:** Emma M. Coomans, Deborah N. Schoonhoven, Hayel Tuncel, Sander C. J. Verfaillie, Emma E. Wolters, Ronald Boellaard, Rik Ossenkoppele, Anouk den Braber, Wiep Scheper, Patrick Schober, Steven P. Sweeney, J. Michael Ryan, Robert C. Schuit, Albert D. Windhorst, Frederik Barkhof, Philip Scheltens, Sandeep S. V. Golla, Arjan Hillebrand, Alida A. Gouw, Bart N. M. van Berckel

**Affiliations:** 1 Department of Radiology & Nuclear Medicine, Amsterdam Neuroscience, Vrije Universiteit Amsterdam, Amsterdam UMC, Amsterdam, The Netherlands; 2 Alzheimer Center Amsterdam, Department of Neurology, Amsterdam Neuroscience, Vrije Universiteit Amsterdam, Amsterdam UMC, Amsterdam, The Netherlands; 3 Department of Clinical Neurophysiology and MEG Center, Department of Neurology, Amsterdam Neuroscience, Vrije Universiteit Amsterdam, Amsterdam UMC, Amsterdam, The Netherlands; 4 Clinical Memory Research Unit, Lund University, Lund, Sweden; 5 Department of Clinical Genetics, Amsterdam Neuroscience, Amsterdam UMC, Amsterdam, The Netherlands; 6 Center for Neurogenomics and Cognitive Research, Department of Functional Genomics, Faculty of Science, Vrije Universiteit, Amsterdam, The Netherlands; 7 Department of Anaesthesiology, Vrije Universiteit Amsterdam, Amsterdam UMC, Amsterdam, The Netherlands; 8 Rodin Therapeutics Inc., Cambridge, MA USA; 9 UCL Institutes of Neurology and Healthcare Engineering, London, UK

**Keywords:** Alzheimer, Tau, Synaptic density, Synaptic function, PET, MEG

## Abstract

**Background:**

The mechanism of synaptic loss in Alzheimer’s disease is poorly understood and may be associated with tau pathology. In this combined positron emission tomography (PET) and magnetoencephalography (MEG) study, we aimed to investigate spatial associations between regional tau pathology ([^18^F]flortaucipir PET), synaptic density (synaptic vesicle 2A [^11^C]UCB-J PET) and synaptic function (MEG) in Alzheimer’s disease.

**Methods:**

Seven amyloid-positive Alzheimer’s disease subjects from the Amsterdam Dementia Cohort underwent dynamic 130-min [^18^F]flortaucipir PET, dynamic 60-min [^11^C]UCB-J PET with arterial sampling and 2 × 5-min resting-state MEG measurement. [^18^F]flortaucipir- and [^11^C]UCB-J-specific binding (binding potential, BP_ND_) and MEG spectral measures (relative delta, theta and alpha power; broadband power; and peak frequency) were assessed in cortical brain regions of interest. Associations between regional [^18^F]flortaucipir BP_ND_, [^11^C]UCB-J BP_ND_ and MEG spectral measures were assessed using Spearman correlations and generalized estimating equation models.

**Results:**

Across subjects, higher regional [^18^F]flortaucipir uptake was associated with lower [^11^C]UCB-J uptake. Within subjects, the association between [^11^C]UCB-J and [^18^F]flortaucipir depended on within-subject neocortical tau load; negative associations were observed when neocortical tau load was high, gradually changing into opposite patterns with decreasing neocortical tau burden. Both higher [^18^F]flortaucipir and lower [^11^C]UCB-J uptake were associated with altered synaptic function, indicative of slowing of oscillatory activity, most pronounced in the occipital lobe.

**Conclusions:**

These results indicate that in Alzheimer’s disease, tau pathology is closely associated with reduced synaptic density and synaptic dysfunction.

**Supplementary Information:**

The online version contains supplementary material available at 10.1186/s13195-021-00772-0.

## Introduction

Synaptic and neuronal loss, combined with ß-amyloid (Aß) plaques and neurofibrillary tangles (NFT), comprise the main pathological characteristics of Alzheimer’s disease (AD) [1]. Clinico-pathological studies have indicated synaptic loss as the major structural correlate of cognitive decline in AD [2, 3]. In patients with AD, previous positron emission tomography (PET) studies have shown that the degree and spatial distribution of in vivo tau pathology closely correlates with disease severity, clinical symptoms and AD phenotypes [4–7]. This makes tau a likely candidate to be associated with synaptic loss in AD. Indeed, neuropathological evidence suggests that pathological tau isoforms localize at the synapse [8–12], where it can have devastating effects on synaptic structure and function [13, 14]. However, to date information describing the link between tau pathology and synaptic loss in the human brain is mainly limited to post-mortem studies.

The novel synaptic [^11^C]UCB-J PET radiotracer has nanomolar affinity for the presynaptic vesicle glycoprotein 2A (SV2A) [15], providing a direct in vivo measure of synaptic density [16]. Recent studies in patients with AD have observed strongest reductions in SV2A radiotracer-binding in the hippocampus [17–19], a key region for structural synaptic degeneration in AD [20]. Furthermore, a recent PET study observed an association between decreased SV2A-binding and increased tau-binding in the medial temporal lobe in patients with amnestic mild cognitive impairment [21].

While [^11^C]UCB-J PET is a measure of synaptic density, magnetoencephalography (MEG) provides a direct measure of synaptic activity as it measures the magnetic fields generated by post-synaptic currents, allowing measurement of synaptic (dys)function in vivo with high temporal resolution [22, 23]. Neuronal oscillatory activity is strongly linked to the balance between synaptic currents from inhibitory interneurons and excitatory pyramidal cells [24]. Typically, healthy neuronal circuits are in an equilibrium between synaptic excitation and inhibition [24], which is critical for the formation of oscillations [25]. Disturbances in this balance can disrupt oscillatory activity and thereby cause dysfunction. Interestingly, in a computational modelling study, alterations to the excitation-inhibition balance led to the onset of AD hallmarks such as oscillatory slowing and loss of absolute broadband power [26]. Measures of synaptic activity and function can be obtained using spectral MEG metrics such as relative power, absolute (broadband) power and peak frequency. Previous MEG studies in AD have described a global cortical slowing of neuronal oscillatory activity, as reflected by higher relative power in the lower (delta and theta) frequency bands, lower relative power in the higher (alpha and beta) frequency bands and lower peak frequency [27].

It is currently unknown how and to what extent tau pathology is associated with changes in synaptic density (structure) and activity (function) in patients with AD. In the present proof-of-concept study, we investigated the associations between tau pathology ([^18^F]flortaucipir PET), synaptic density ([^11^C]UCB-J PET) and oscillatory activity (MEG) in subjects with AD. We hypothesized that regional higher [^18^F]flortaucipir uptake is associated with lower [^11^C]UCB-J uptake. In addition, we hypothesized that regional higher [^18^F]flortaucipir uptake and lower [^11^C]UCB-J uptake are associated with lower absolute broadband power and measures that are indicative of slowing of oscillatory activity (i.e. higher relative delta and theta power, lower relative alpha power and lower peak frequency).

## Methods

### Subjects

Seven subjects with a clinical diagnosis of probable AD [28] were included from the Amsterdam Dementia Cohort after extensive clinical screening [29]. All subjects had positive biomarkers for Aß pathology by means of cerebrospinal fluid (CSF) Aß_1–42_ and/or visual read of Aß-PET ([^11^C]PiB, [^18^F]florbetaben, or [^18^F]flutemetamol). Exclusion criteria were a Mini-Mental State Examination (MMSE) score of < 18, magnetic resonance imaging (MRI) medial temporal atrophy score of ≥ 3, blood haemoglobin levels ≤ 8 for males and ≤ 7 for females, presence of major psychiatric or neurological disorders other than AD, severe claustrophobia and the presence of intracorporal devices interfering with MEG signals. The BEBO Foundation Medical Ethics Committee (Assen, The Netherlands) and the local Institutional Review Board of the VUmc (Amsterdam, The Netherlands) approved the study. All subjects provided written informed consent prior to the study.

### PET acquisition procedures

All subjects underwent two 60-min dynamic [^11^C]UCB-J PET scans within ± 28 days as they additionally took part in a kinetic modelling study to assess [^11^C]UCB-J test-retest variability [30]. For each subject, the first [^11^C]UCB-J PET was used for data analysis. In addition, all subjects underwent a 130-min dynamic [^18^F]flortaucipir PET scan with a median time lag between [^11^C]UCB-J and [^18^F]flortaucipir PET of 95 days (interquartile range 133 days). All PET scans were acquired on an Ingenuity TF PET/CT scanner (Philips Medical Systems, Best, The Netherlands), preceded by a low-dose CT scan for attenuation correction purposes. [^11^C]UCB-J and [^18^F]flortaucipir radiotracer syntheses were performed on-site [15, 31]. Prior to PET scanning, all subjects received a venous cannula for tracer injection. For the [^11^C]UCB-J PET scan, an additional radial artery cannula for arterial sampling was inserted in order to obtain a metabolite corrected plasma input function for full quantification of [^11^C]UCB-J [30]. Dynamic 60-min [^11^C]UCB-J PET data were acquired immediately following a bolus injection of 329 ± 31 MBq [^11^C]UCB-J. Each [^11^C]UCB-J PET dataset consisted of 19 frames [30]. [^18^F]flortaucipir PET data were acquired immediately following a bolus injection of 229 ± 12 MBq [^18^F]flortaucipir as described previously [31]. All PET data were 3D reconstructed using the vendor provided image reconstruction method (RAMLA) with a matrix size of 128 × 128 × 90 and a final voxel size of 2 × 2 × 2 m^3^. All standard corrections for attenuation, scatter, randoms, decay and dead time were performed.

### MRI acquisition procedures

Three-dimensional T1-weighted MRI scans were acquired for all patients on a 3.0T Ingenuity TF PET/MR system (Philips Medical Systems, Best, The Netherlands) within a maximum of 2 and 6 months from [^11^C]UCB-J and [^18^F]flortaucipir PET, respectively. None of the subjects had significant structural abnormalities that may have interfered with the analysis of the PET scans.

### MEG acquisition procedures

Six out of seven subjects additionally underwent MEG measurement within 6 months from the [^18^F]flortaucipir PET scan (median 125 days, interquartile range 84 days) and within 9 months from the [^11^C]UCB-J scan (median 170 days, interquartile range 217 days). MEG-data were acquired by means of a 306-channel whole-head MEG-system (Elekta Neuromag Oy, Helsinki, Finland), while subjects were supine in a magnetically shielded room (VacuumSchmelze GmbH, Hanua, Germany). Two 5-min eyes-closed resting-state recordings were acquired. Subjects were instructed to relax and stay awake. Several times during the recordings, subjects were instructed to briefly open their eyes. Magnetic fields were recorded with a sample frequency of 1250 Hz, using an online anti-aliasing filter of 410 Hz and a high-pass filter of 0.1 Hz. The subjects’ head position in relation to the MEG sensors was continuously recorded using signals from five head-localization coils.

### PET and MEG imaging analyses

Structural 3D T1-weighted MR images were co-registered to the PET images using Vinci software. For each subject, regions of interests (ROIs) were defined on the co-registered MRI scan with the Hammers template [32], which is incorporated in PVElab, a software programme that uses a probability map of 60 delineated (grey matter) ROIs that has been validated previously [33]. These ROIs were then superimposed onto the dynamic PET scans to extract regional time activity curves (TACs). [^11^C]UCB-J data were analysed using a plasma input reversible single tissue compartmental model with blood volume correction (1T2k_V_B_) to fit the regional TACs, in order to obtain regional volumes of distribution (V_T_, the tissue-to-plasma concentration ratio at equilibrium) [34, 35]. Binding potential (BP_ND_) was calculated for each ROI using a manually delineated centrum semi-ovale as reference region (BP_ND_ = V_T_ROI_/V_T_reference_−1) [15, 17]. Centrum semi-ovale V_T_ was used as the reference region for quantification of [^11^C]UCB-J [15, 30]. In addition, only for visualization purposes, parametric SRTM2-derived BP_ND_ images were generated. For [^18^F]flortaucipir data, receptor parametric mapping (RPM)-derived BP_ND_, with grey matter cerebellum as reference region, were obtained, which is a validated method to quantify tau load [36]. A partial volume correction method that combines Van Cittert iterative deconvolution methods (IDM) with highly constrained back projection (HYPR) was applied to the [^18^F]flortaucipir BP_ND_ data for a better quantification of the dynamic PET signal [37]. Partial volume correction was not applied to the [^11^C]UCB-J data, because there is currently no validated method available for that purpose.

The PET-co-registered MRI scan and the subject-space Hammers template were additionally used in MEG analyses. Raw MEG-data were visually inspected for malfunctioning and noisy channels, which were subsequently removed, after which the temporal extension of Signal Space Separation (tSSS) in MaxFilter software (Elekta Neuromag Oy, version 2.2.12) [38] was applied in order to remove artefacts from the data [39]. Data were subsequently filtered in the 0.5–48 Hz band using MaxFilter software. Each subjects’ MEG data were co-registered with the PET-coregistered MRI using surface matching, after which the same transformation was applied to the subject-space Hammers template. In order to reconstruct neuronal activity at source level, an atlas-based beamforming approach was applied [40], using the centroids [41] of the 67 parcels in the Hammers template. For each of these centroid voxels (i.e. virtual electrodes), time-series of neuronal activity were reconstructed by projecting the sensor signals to source space. Broadband data (0.5–48 Hz) were used for the estimation of the beamformer weights, in order to avoid overestimation of covariance between channels [42], as well as a unity noise covariance matrix, a spherical head model (fitted to the scalp surface as extracted from the MRI) and an equivalent current dipole as source model. On average 300 s of data (range 297–303 s) was used for the estimation of the data covariance matrix, which was regularized using singular value truncation with the default setting of 1e−06 times the maximum singular value. The optimum orientation of the equivalent current dipole was found using singular value decomposition [43]. The broadband sensor-level data were subsequently and sequentially projected through the normalized beamformer weights [44], resulting in a time-series for each voxel. The time-series for these voxels were subsequently used for further analysis. For each subject, 10 non-overlapping, artefact-free, eyes-closed, down-sampled epochs of 4096 samples (13.1072 s) were selected, based on careful visual inspection. Inspection and further analysis was done using in-house software Brainwave (version 0.9.152.12.26, available from http://home.kpn.nl/stam7883/brainwave.html). A discrete Fast Fourier transform was applied to the time series of each parcel (*n* = 67) in order to estimate absolute broadband (0.5–48 Hz) power, which is the sum of power across all bands; the relative power (power for a band divided by the sum of power for all bands) in three canonical MEG frequency bands (delta (0.5–4 Hz), theta (4–8 Hz) and alpha (8–3 Hz)); and the peak-frequency, defined here as the dominant frequency in the 4–13 Hz range [26, 27]. Per subject, these spectral measures were averaged over epochs.

Mean [^11^C]UCB-J and [^18^F]flortaucipir BP_ND_ were extracted within, and mean MEG spectral measures were used for 12 a priori selected bilateral cortical ROIs [45, 46] of the Hammers template, namely those in the temporal (middle and inferior temporal gyri; superior temporal gyrus, posterior temporal lobe), parietal (superior parietal gyrus, inferolateral parietal lobe), occipital (cuneus, lateral occipital lobe) and frontal (middle frontal gyrus, superior frontal gyrus, inferior frontal gyrus, orbitofrontal gyrus, gyrus rectus) lobes. To enable exploration of associations with cognition across subjects, we computed volume-weighted averages of [^11^C]UCB-J BP_ND_ and [^18^F]flortaucipir BP_ND_ and averages for MEG spectral measures across ROIs.

### Statistical analyses

We performed two sets of analyses. Firstly, Spearman correlation models (IBM SPSS Statistics, version 26) were performed across ROIs and subjects, as well as across ROIs within subjects (separate analyses), to investigate associations between [^18^F]flortaucipir BP_ND_ and [^11^C]UCB-J BP_ND_. To compensate for inter-regional dependencies within each subject, significant associations were subsequently assessed using generalized estimating equation (GEE) with [^18^F]flortaucipir BP_ND_ as the predictor, [^11^C]UCB-J BP_ND_ as the outcome variable, and each subject as the repeated measure, using an exchangeable working correlation matrix and a robust estimator.

In a second set of analyses, associations between [^18^F]flortaucipir BP_ND_ or [^11^C]UCB-J BP_ND_ and MEG spectral measures were investigated across subjects. Analyses were performed separately for each brain lobe, as physiological brain oscillatory characteristics are region-dependent [27, 47]. For example, occipital regions are the main cortical generators of the dominant oscillatory activity in the alpha frequency range (8–13 Hz) during resting-state conditions [48], whereas alpha activity in frontal regions is low in general. Furthermore, the frontal regions in MEG have a lower signal-to-noise ratio. We therefore decided to stratify the analyses by brain lobes instead of performing one analysis encapsulating all brain lobes. Spearman correlations were performed across ROIs and subjects (for each brain lobe separately), and significant associations were subsequently assessed using GEE analyses (with [^18^F]flortaucipir BP_ND_ or [^11^C]UCB-J BP_ND_ as the predictor and MEG spectral measures as the outcome) to correct for possible inter-regional dependencies. A *p* < 0.05 was considered statistically significant.

Finally, to explore whether each imaging modality was associated with cognition, we performed Spearman correlation analyses between MMSE and average [^18^F]flortaucipir BP_ND_, [^11^C]UCB-J BP_ND_ and MEG spectral measures. We selected relative alpha power based on previous work [49]. Additionally, we added absolute broadband power as it contains all frequency bands. Because relative alpha power is most prominently observed in the occipital lobe [48], we used occipital relative alpha power and absolute broadband power for these analyses.

## Results

**Fig. 1:**
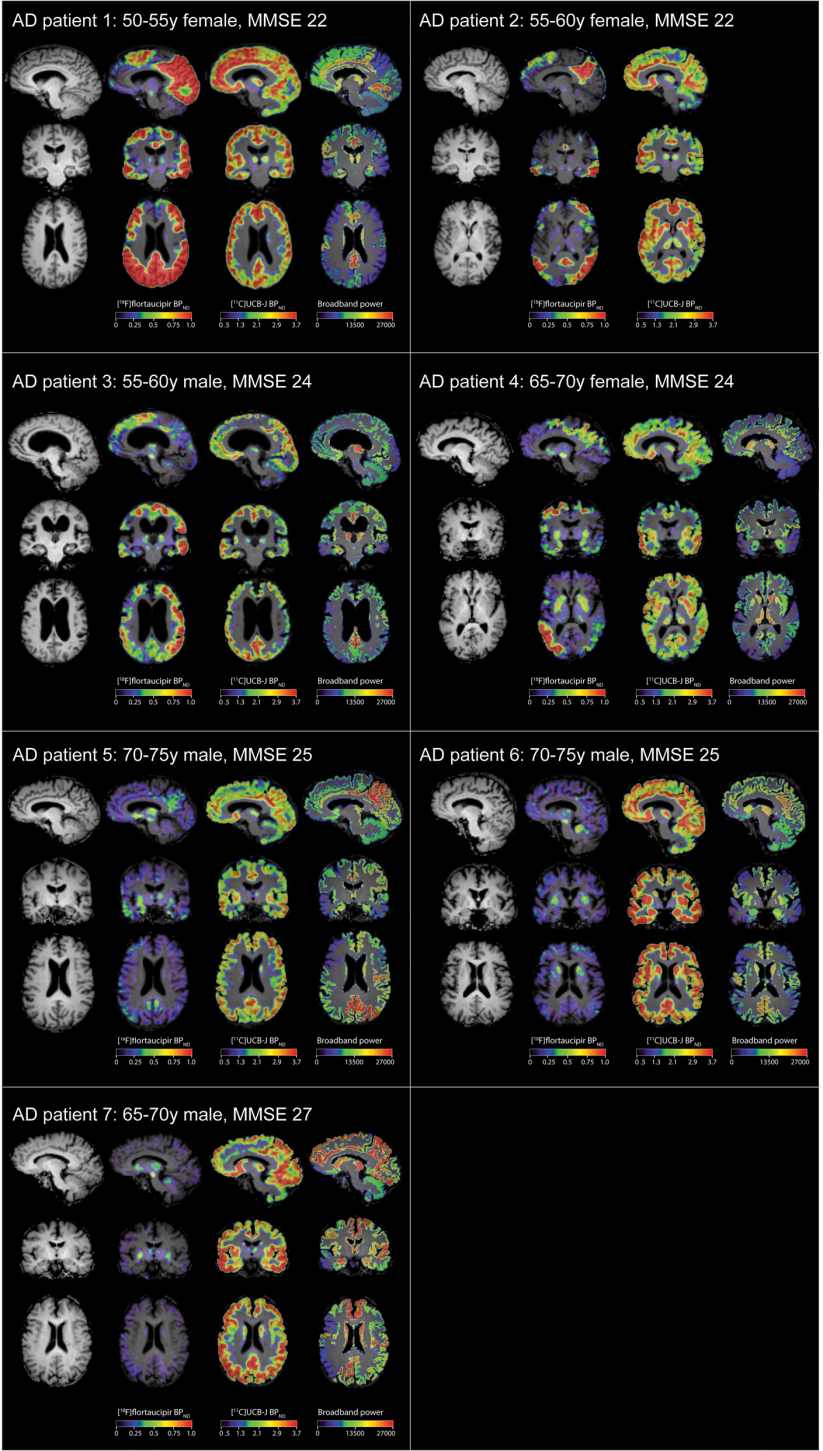
Regional distributions of [^18^F]flortaucipir BP_ND_, [^11^C]UCB-J BP_ND_ and MEG absolute broadband power of each subject. Shown are parametric images of RPM-derived [^18^F]flortaucipir BP_ND_, parametric images of SRTM2-derived [^11^C]UCB-J BP_ND_ and MEG broadband power in all ROIs from the Hammers template. The order of patients corresponds to the order of average [^18^F]flortaucipir BP_ND_ (high to low)

Seven Aβ-biomarker supported AD dementia subjects (3 female; 4 male) with a mean age of 64.3 ± 8.2 years and MMSE score of 24.1 ± 1.8 were included. Figure 1 presents the [^18^F]flortaucipir PET and corresponding co-registered [^11^C]UCB-J PET and MEG broadband power plot for each subject, with subjects ordered from high to low values of neocortical [^18^F]flortaucipir BP_ND_. Upon visual inspection, brain regions with high [^18^F]flortaucipir BP_ND_ corresponded closely with the regions that showed most prominent decreases in [^11^C]UCB-J BP_ND_, mainly observed in temporoparietal regions. Furthermore, low MEG broadband power was observed in temporal regions for all subjects.

### Associations between [^18^F]flortaucipir BP_ND_ and [^11^C]UCB-J BP_ND_

**Fig. 2:**
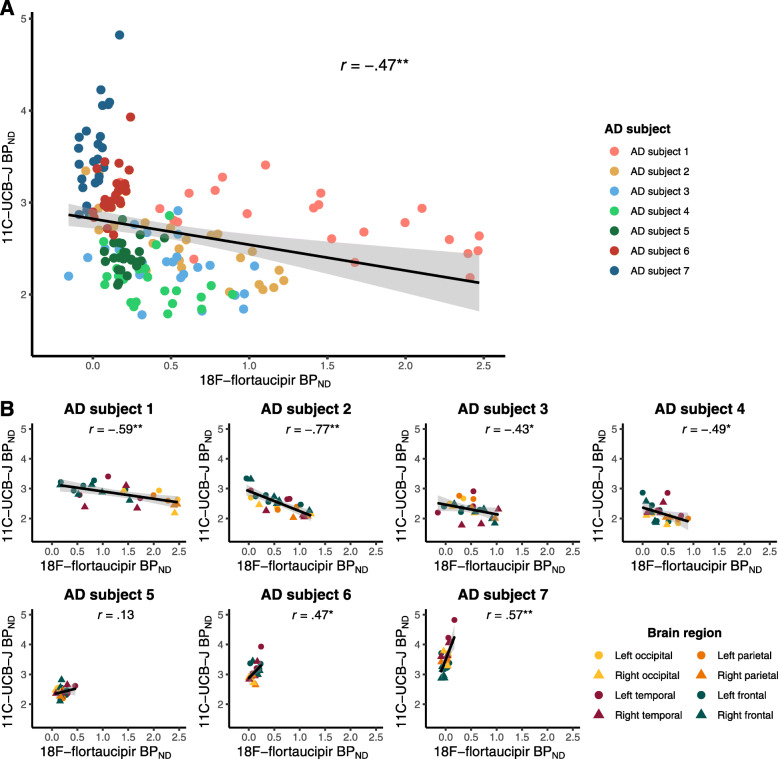
Across-subject correlations (**a**) and within-subject correlations (**b**) between regional [^18^F]flortaucipir BP_ND_ (*x*-axis) and [^11^C]UCB-J BP_ND_ (*y*-axis). [^18^F]flortaucipir BP_ND_ was partial volume corrected. **a** Colours represent different AD subjects; multiple ROIs per AD subject are shown. The black line represents the correlation line across all subjects and ROIs. **b** Colours represent cortical ROIs from the temporal, parietal, frontal and occipital brain lobes with triangles representing right-hemisphere regions and circles representing left-hemisphere regions. *p < 0.05; **p < 0.01

First, we examined the association between tau pathology and synaptic density. Across subjects and ROIs, higher [^18^F]flortaucipir BP_ND_ was associated with lower [^11^C]UCB-J BP_ND_ (*r* = −0.47, *p* < 0.001) (Fig. 2a, with AD subject numbers analogous to Fig. 1). This association remained significant after correcting for dependency of ROIs within subjects using GEE analyses (*β* = −0.30, *p* < 0.001). Within each subject separately, in subjects with the highest neocortical tau levels, higher [^18^F]flortaucipir BP_ND_ was associated with lower [^11^C]UCB-J BP_ND_ across ROIs (AD subjects 1–4: *r* = −0.59, *p* = 0.003; *r* = −0.77, *p* < 0.001; *r* = −0.43, *p* = 0.04; and *r* = −0.49, *p* = 0.02, respectively) (Fig. 2b). In contrast, within subjects with relatively low neocortical tau levels, higher [^18^F]flortaucipir BP_ND_ was associated with higher [^11^C]UCB-J BP_ND_ across ROIs (AD subject 6–7: *r* = 0.47, *p* = 0.02, and *r* = 0.57, *p* = 0.004, respectively) (Fig. 2b). Associations remained unchanged when using the second [^11^C]UCB-J PET scan instead of the first [^11^C]UCBJ PET scan.

### Associations between [^18^F]flortaucipir BP_ND_, [^11^C]UCB-J BP_ND_ and MEG spectral measures

**Fig. 3:**
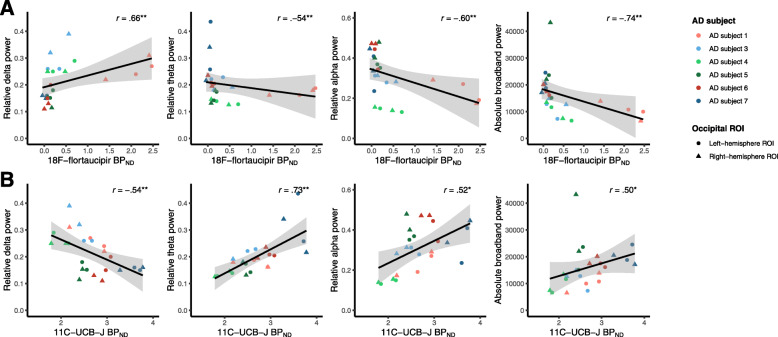
Across-subject correlations between regional [^18^F]flortaucipir or [^11^C]UCB-J and MEG spectral measures in the occipital lobe. Shown are scatterplots of the correlations between **a** regional [^18^F]flortaucipir and MEG spectral measures and **b** regional [^11^C]UCB-J and MEG spectral measures, in the occipital lobe (Spearman correlation coefficients). Colours represent different AD subjects; multiple occipital regions per subject are shown. Triangles represent right-hemisphere regions, circles left-hemisphere regions. Upon correcting for dependency of multiple ROIs per subject using GEE analyses, all correlations remained significant, except for the correlations between [^18^F]flortaucipir BP_ND_ with relative theta power and between [^11^C]UCB-J BP_ND_ with absolute broadband power (*p* = 0.27 and *p* = 0.08 respectively). *p < 0.05; **p < 0.01

Second, we examined the association between tau pathology and synaptic function. Table 1 shows all Spearman correlation coefficients. Higher regional [^18^F]flortaucipir BP_ND_ was associated with lower relative alpha power (occipital: *r* = −0.60, *p* = 0.002; parietal: *r* = −0.48 *p* = 0.017), lower relative theta power (occipital: *r* = −0.54, *p* = 0.007) and higher relative delta power (occipital: *r* = 0.66, *p* = 0.001) (Table 1 and Fig. 3a, with AD subject numbers analogous to Fig. 1), most markedly across regions in the occipital lobe. Across regions in the frontal lobe, an opposite association with relative delta power was observed (*r* = −0.36, *p* = 0.005). Furthermore, higher [^18^F]flortaucipir BP_ND_ was associated with lower broadband power across all brain lobes (occipital: *r* = −0.74, *p* < 0.001; temporal: *r* = −0.51, *p* = 0.001; parietal: *r* = −0.54, *p* = 0.006; frontal: *r* = −0.50, *p* < 0.001). No associations were observed with peak frequency. Scatterplots for the associations in the temporal, parietal and frontal lobe are shown in Supplementary Figure 1–3. After correcting for dependency of ROIs within subjects using GEE analyses, the associations with relative delta power in the occipital and frontal lobe, relative alpha power in the occipital lobe, and all associations with broadband power remained significant (highlighted in colour in Table 1; for GEE beta’s and *p*-values, see Table 2).

**Table 1:**
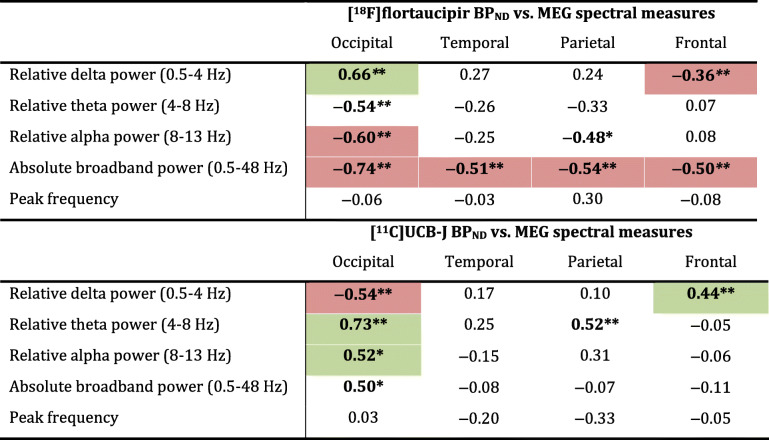
Across-subject Spearman correlations between regional [^18^F]flortaucipir BP_ND_ or [^11^C]UCB-J BP_ND_ and MEG power spectral measurements

Spearman’s rho correlation coefficients are shown for associations between [^18^F]flortaucipir BP_ND_ (partial volume corrected) or [^11^C]UCB-J BP_ND_ with MEG spectral measures (relative delta, theta and alpha power, absolute broadband power and peak frequency). Associations that remained significant upon correcting for multiple ROIs per subject using GEE analyses are highlighted in colour (green: positive association; red: negative association)

*p < 0.05; ** *p* < 0.01

**Table 2:**
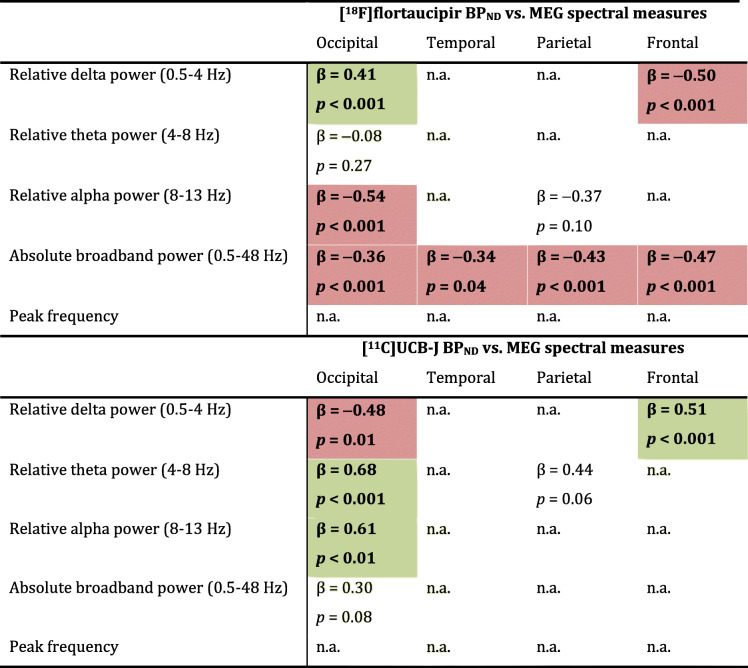
Across-subject generalized estimating equations between regional [^18^F]flortaucipir BP_ND_ or [^11^C]UCB-J BP_ND_ and MEG spectral measurements

We only computed generalized estimating equations (GEE) for those associations that were significant when assessed with Spearman correlations (Table 1). Standardized beta (β) and *p* values from GEE analyses between [^18^F]flortaucipir BP_ND_ (partial volume corrected) or [^11^C]UCB-J and MEG spectral measures (relative delta, theta and alpha power; absolute broadband power; and peak frequency) are shown. In green, significantly positive associations are shown; in red, significantly negative associations are shown

Third, we examined the association between synaptic density and synaptic function. Higher regional [^11^C]UCB-J BP_ND_ was associated with higher relative alpha (occipital: *r* = 0.52, *p* = 0.010), higher relative theta power (occipital; *r* = 0.73, *p* < 0.001; parietal: *r* = 0.52; *p* = 0.005) and lower relative delta power (occipital: *r* = −0.54; *p* = 0.007) (Table 1 and Fig. 3b). Again, across regions in the frontal lobe, an opposite association was observed with relative delta power (*r* = 0.44, *p* < 0.001). Finally, higher regional [^11^C]UCB-J BP_ND_ was associated with higher broadband power in the occipital lobe (*r* = 0.50, *p* = 0.013). Again, no associations were observed with peak frequency. Scatterplots for the associations in the temporal, parietal and frontal lobe are shown in Supplementary Figure 1–3. After correcting for dependency of ROIs within subjects using GEE analyses, the associations with occipital relative delta, theta and alpha power, and frontal delta power, remained significant, while occipital broadband power and parietal relative theta power lost significance (highlighted in colour in Table 1; for GEE beta’s and *p*-values, see Table 2).

### Exploratory analyses

Finally, we explored the association between each imaging modality and cognition. MMSE was strongly associated with [^18^F]flortaucipir BP_ND_ (*r* = − 0.97; *p* < 0.001). MMSE was not associated with [^11^C]UCB-J BP_ND_ (*r* = 0.39; *p* = 0.39), MEG occipital absolute broadband power (*r* = 0.79, *p* = 0.06), or occipital alpha power (*r* = 0.71; *p* = 0.12) (Supplementary Figure 4).

## Discussion

In the present study, we examined the in vivo associations between tau pathology, synaptic density and synaptic function in AD. In line with our hypotheses, higher [^18^F]flortaucipir uptake was associated with lower [^11^C]UCB-J uptake across subjects. Moreover, both higher [^18^F]flortaucipir and lower [^11^C]UCB-J uptake were associated with altered synaptic function in the occipital lobe. Furthermore, higher [^18^F]flortaucipir binding was associated to loss of absolute broadband power across all brain lobes. Finally, we observed that within-subject regional associations between [^18^F]flortaucipir and [^11^C]UCB-J uptake depended on the degree of neocortical tau pathology. Our results provide in vivo support for an interaction between tau pathology and the synapse.

Across subjects, regional higher tau pathology was associated with synaptic loss. This negative association across regions was particularly strong for subjects with substantial neocortical tau burden. This is in line with post-mortem human brain studies that showed lower levels of synaptic protein expression in NFT-containing neurons compared to neurons without NFT [11, 12, 50]. We furthermore observed a high level of spatial overlap between higher [^18^F]flortaucipir and lower [^11^C]UCB-J binding (Fig. 1). In line with a previous study from Vanhaute and others [21], the pattern of tau pathology was slightly more widespread and pronounced compared to the pattern of reduced synaptic density, which corresponds with a conceptual model that suggests tau to precede and potentially drive structural degeneration and loss of synaptic function [51].

In our study, we provide in vivo evidence for an association between tau pathology and altered synaptic function. Higher [^18^F]flortaucipir uptake was associated with higher relative oscillatory power in lower frequencies (delta) and lower relative power in higher frequencies (alpha). These findings are suggestive of oscillatory slowing, which is typically characterized by higher relative power in the delta and theta frequency bands, lower relative power in the alpha and beta bands and lower peak frequency. However, we did not find associations with peak frequency, possibly due to the small sample size. In addition, a negative association with relative theta power was observed, although it did not survive correction for dependency of ROIs within subjects. Our findings were most pronounced in the occipital lobe, where the posterior dominant rhythm during a resting state eyes closed condition normally is present. This is in agreement with previous quantitative MEG/EEG studies reporting slowing of oscillatory activity most frequently in parietal, occipital and temporal areas in AD [27, 52], and has previously been linked to synaptic dysfunction [53]. Indeed, dysfunction in both excitatory or inhibitory synaptic transmission has been proposed as root causes for the altered brain function in AD [54, 55].

In the present study, we used absolute broadband power as a reflection of overall neuronal activity [26, 56], which has been shown to decrease with increasing neurodegeneration [26]. We found that higher tau load was consistently associated with lower broadband power in all brain lobes, suggesting that with increasing tau load, total neuronal activity declines. Overall, our results suggest that higher tau pathology is correlated with alterations in synaptic activity, as expressions of synaptic degeneration or dysfunction.

We found opposite patterns between [^11^C]UCB-J uptake and MEG relative power measures as compared to [^18^F]flortaucipir binding. More specifically, we observed that lower synaptic density was associated with higher relative delta power, and with lower relative theta and lower alpha power, particularly in the occipital lobe. We only found an association between higher [^11^C]UCB-J uptake and higher absolute broadband power in the occipital lobe; however, this association lost significance upon correcting for dependency of ROIs within subjects. The findings in the occipital lobe carefully suggest that regional loss of synapses is related to more oscillatory slowing and decline in neuronal activity.

Counterintuitive effects were observed in the frontal lobe for both [^18^F]flortaucipir and [^11^C]UCB-J uptake in relation to relative delta power. The frontal regions in MEG are known to have low signal to noise ratio. We postulate that the counterintuitive results could have been caused by the use of relative instead of absolute values of band power, where relative delta power is artificially high (i.e. even with low broadband power, the sum of all relative band power values is 1 by definition). Another explanation may be the high prevalence of artefacts (e.g. due to eye movements) in the frontal regions, even after using source reconstructed data. Indeed, a previous MEG study also showed high frontal relative delta power in both AD and healthy subjects [57] without differences between groups. These associations should therefore be interpreted with caution. However, recent findings by Ranasinghe and colleagues do suggest that the frontal delta band may contain clinically relevant information regarding the role of tau pathology in Alzheimer’s disease [49].

Ranasinghe and colleagues evaluated the association between [^18^F]flortaucipir PET and an MEG measure of functional connectivity between neuronal populations [49]. Direct comparisons should be made with caution, as in our study we used spectral measures within neuronal populations (i.e. relative power) while Ranasinghe et al. used a measure of functional connectivity between neuronal populations (i.e. imaginary coherence). They observed that [^18^F]flortaucipir uptake strongly co-localized with alpha band hypo-synchrony and delta-theta band hyper-synchrony. Spatial colocalization between [^18^F]flortaucipir uptake and functional connectivity was most pronounced in temporoparietal regions, while our results were most pronounced in the occipital lobe. Additionally, Ranasinghe et al. identified the bilateral occipital regions among the key regions that show alpha hypo-synchrony in AD compared to controls. Since functional connectivity measures reflect different aspects of brain function than spectral measures, this divergence in regional specificity for correlations between [^18^F]flortaucipir uptake and different aspects of neuronal functioning could be further investigated in future studies. Nevertheless, the similarities in frequency-specific MEG associations with tau pathology between this and our study are promising.

Although we performed a cross-sectional study, and causation cannot be inferred, our findings may reflect synaptic loss as a consequence of tau pathology. This is corroborated by our within-subject results which showed that the association between tau pathology and synaptic density depended on the level of neocortical tau load. More specifically, positive associations were observed when neocortical tau load was low, gradually changing into negative associations with increasing neocortical tau burden. The AD subjects showing positive associations scored relatively high on the MMSE and had a relatively high age compared to the other AD subjects (see Fig. 1), though further research is needed to look into this pattern in more detail. Importantly, all AD subjects in the current study showed evidence for the presence of Aβ pathology. The positive within-subject association between tau pathology and synaptic density in low-tau subjects could be driven by the presence of Aβ pathology without substantial tau pathology, while the negative within-subject association in high-tau subjects could be driven by tau pathology. It has also previously been reported that differential phases in functional connectivity may be present in relation to AD pathology, with a phase of hyper-connectivity in Aβ-positive individuals when neocortical tau was low, and a phase of hypo-connectivity when neocortical tau was high [58]. In line with these findings, our results may also indicate a phase of regional synaptic upregulation in the presence of predominantly Aβ pathology, gradually resulting in a phase of regional synaptic loss with additional increased neocortical tau pathology, suggesting Aβ and tau to act in concert in synaptic degeneration.

Increasing evidence suggests that the spreading of tau pathology in AD may occur trans-synaptically [59], making synapses a particularly vulnerable site for tau-related structural damage. In previous in vitro and AD mouse model studies, enhancement of synaptic activity has been reported to stimulate the release and spread of endogenous tau and to accelerate tauopathy [60–62]. Other studies showed that pathological tau in turn reduced the activity of functionally connected neurons as measured with intracellular recordings, and perturbed the synchronous synaptic activity pattern of the network [63, 64]. In line with current hypotheses from different in vivo imaging modalities [26, 58, 65, 66], Aβ deposition in the early phases of AD may initially cause a phase of hyperactivity, which could in turn enhance or stimulate regional tau deposition. In later phases of AD, this process may lead to more widespread tau deposition, neuronal damage and synaptic loss and eventually cognitive decline. Our within-subject differential associations between tau pathology and synaptic density may add support to these hypotheses. However, although we observed an association between tau pathology and MMSE, MMSE was not associated with synaptic density in our sample. This may be related to our small sample size, and future studies with larger sample sizes are needed to investigate this further.

### Limitations

This proof-of-concept study has a few limitations, with the main limitation being the sample size. Therefore, cautious interpretation of our findings is needed. It must be noted that the dynamic range and extent of synaptic loss needed for [^11^C]UCB-J PET detection is currently unknown. Furthermore, no partial volume correction was applied to the [^11^C]UCB-J data because this has not yet been validated. Importantly, it must be taken into account that the [^18^F]flortaucipir tracer provides a measure for NFT pathology, while most preclinical studies have shown oligomeric tau as the pathological tau isoform at the synapse. In addition, there was a time window of 6–9 month times between PET and MEG measurements. Another limitation to be considered is the use of anticholinergic medication of subjects 1, 3, 4 and 7, which is a known modulator of oscillatory activity. However, previous research suggests that administration of cholinesterase inhibitors *reverses* the slowing of oscillatory activity, i.e. delta and theta rhythms decrease and alpha rhythms increase [67, 68]. All subjects with high tau burden who underwent MEG measurement used anticholinergic medication; therefore, the effects found in this study may have been an underestimation of the true effects. Additionally, because of the small sample size, it was not possible to remove subjects with suboptimal epochs (e.g. those that included possible effects of drowsiness) from the analysis. Recent work suggest though that this may have minimal effect on the spectral measures used in the current study [69]. Also, the postulated loss of absolute broadband power as a measure of neurodegeneration needs to be further confirmed with other methods that reflect overall activity. Notwithstanding, despite a small sample size, we found relatively strong and robust findings between tau pathology, synaptic loss and synaptic (dys)function. We used state-of-the art quantification methods, and our results may contribute to a better understanding of the third AD pathological hallmark—synaptic loss—and may generate new hypotheses about synaptic plasticity in relation to AD pathology.

## Conclusions

Overall, we provide preliminary in vivo evidence for the spatial and frequency-specific association between tau pathology, synaptic loss and synaptic dysfunction. Within-subject associations between tau pathology and synaptic density may depend on the total neocortical tau burden. These results indicate that in Alzheimer’s disease tau pathology is closely associated with affected synaptic density and synaptic function.

## Supplementary Information

**Additional file 1: Figure S1**. Across-subject correlations between regional [^18^F]flortaucipir BP_ND_ or [^11^C]UCB-J BP_ND_ and MEG spectral measures in the temporal lobe. **Figure S2**. Across-subject correlations between regional [^18^F]flortaucipir BP_ND_ or [^11^C]UCB-J BP_ND_ and MEG spectral measures in the parietal lobe. **Figure S3**. Across-subject correlations between regional [^18^F]flortaucipir BP_ND_, or [^11^C]UCB-J BP_ND_, and MEG spectral measures in the frontal lobe. **Figure S4**. Correlations between MMSE and averages of [^18^F]flortaucipir BP_ND_, [^11^C]UCB-J BP_ND_, MEG occipital absolute broadband power and MEG occipital relative alpha power.

## Data Availability

The data used in this study are not publicly available, but may be provided upon reasonable request.

## Abbreviations

Aß: Amyloid- ß
NFT: Neurofibrillary tangles
AD: Alzheimer’s disease
PET: Positron emission tomography
SV2A: Synaptic vesicle glycoprotein 2A
MEG: Magnetoencephalography
CSF: Cerebrospinal fluid
MMSE: Mini-Mental State Examination
MRI: Magnetic resonance imaging
ROI: Region of interest
V_T_: Volumes of distribution
BP_ND_: Binding potential
DVR: Distribution volume ratio
RPM: Receptor parametric mapping
GEE: Generalized estimating equation
