# Supplementary material for: In vivo tau pathology is associated with synaptic loss and altered synaptic function
Source: Alzheimers Res Ther. 2021 Feb 5;13:35. doi: 10.1186/s13195-021-00772-0 (PMC7866464; doi:10.1186/s13195-021-00772-0)
Supplement: Supplementary file 1 — Additional file 1: Figure S1. Across-subject correlations between regional [18F]flortaucipir BPND or [11C]UCB-J BPND and MEG spectral measures in the temporal lobe. Figure S2. Across-subject correlations between regional [18F]flortaucipir BPND or [11C]UCB-J BPND and MEG spectral measures in the parietal lobe. Figure S3. Across-subject correlations between regional [18F]flortaucipir BPND, or [11C]UCB-J BPND, and MEG spectral measures in the frontal lobe. Figure S4. Correlations between MMSE and averages of [18F]flortaucipir BPND, [11C]UCB-J BPND, MEG occipital absolute broadband power and MEG occipital relative alpha power. [file 13195_2021_772_MOESM1_ESM.docx]

**Supplementary Figure 1. Across-subject correlations between regional [^18^F]flortaucipir BP_ND_ or [^11^C]UCB-J BP_ND_ and MEG spectral measures in the temporal lobe**

**
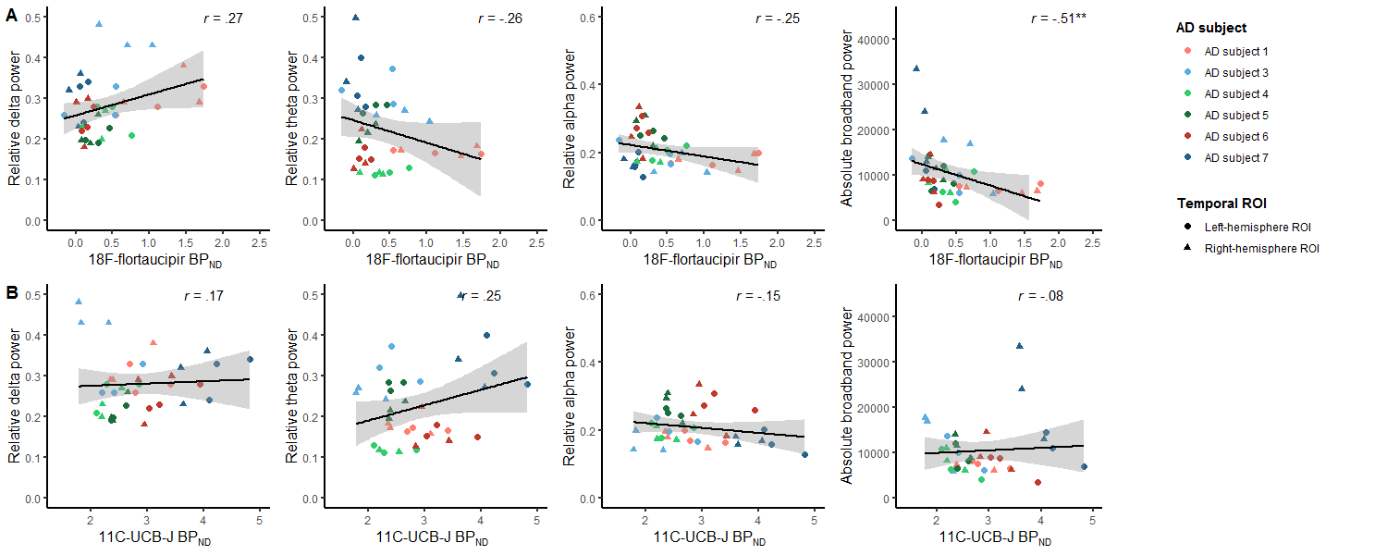
**

Shown are scatterplots of the correlations between (A) regional [^18^F]flortaucipir BP_ND_ and MEG spectral measures and (B) regional [^11^C]UCB-J BP_ND_ and MEG spectral measures, in the temporal lobe (Spearman correlation coefficients). Colours represent different AD subjects, multiple temporal regions per subject are shown. Triangles represent right-hemisphere regions, circles left-hemisphere regions. Upon correcting for dependency of multiple ROIs per subject using GEE analyses, the correlation between absolute broadband power and [^18^F]flortaucipir remained significant. * *p* < 0.05; ** *p* < 0.01

**Supplementary Figure 2. Across-subject correlations between regional [^18^F]flortaucipir BP_ND_ or [^11^C]UCB-J BP_ND_ and MEG spectral measures in the parietal lobe**

**
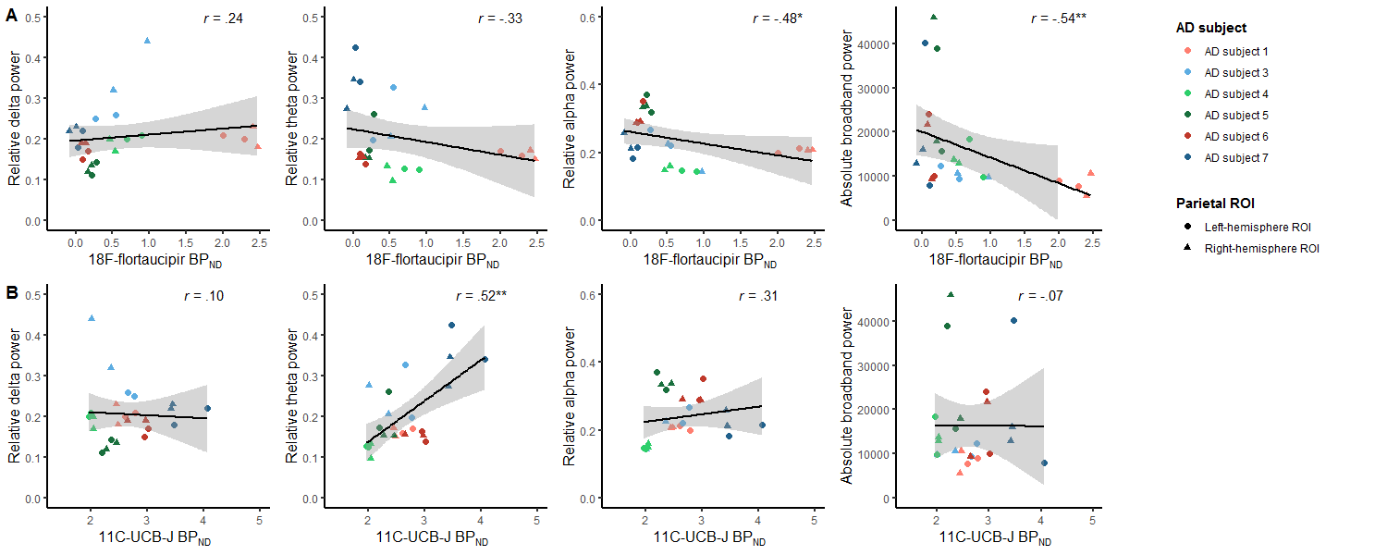
**

Shown are scatterplots of the correlations between (A) regional [^18^F]flortaucipir BP_ND_ and MEG spectral measures and (B) regional [^11^C]UCB-J BP_ND_ and MEG spectral measures, in the parietal lobe (Spearman correlation coefficients). Colours represent different AD subjects, multiple parietal regions per subject are shown. Triangles represent right-hemisphere regions, circles left-hemisphere regions. Upon correcting for dependency of multiple ROIs per subject using GEE analyses, the correlation between relative alpha power and [^18^F]flortaucipir, and relative theta power and [^11^C]UCB-J lost significance (p=0.10 and p=0.06 respectively). * *p* < 0.05; ** *p* < 0.01

**Supplementary Figure 3. Across-subject correlations between regional [^18^F]flortaucipir BP_ND_, or [^11^C]UCB-J BP_ND_, and MEG spectral measures in the frontal lobe**

**
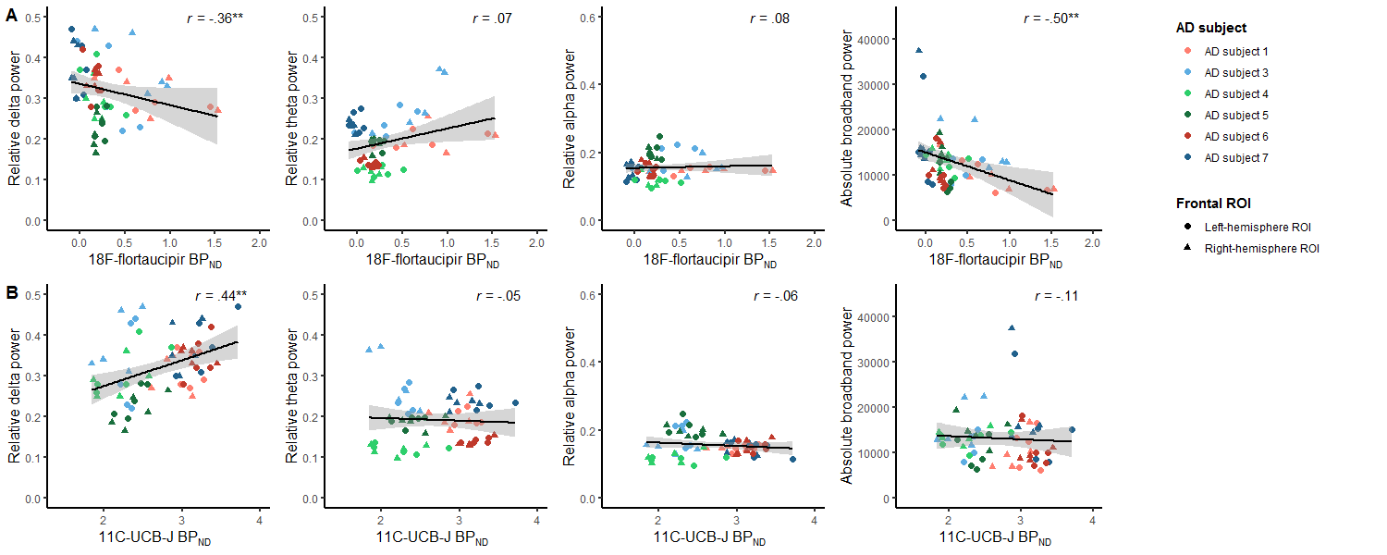
**

Shown are scatterplots of the correlations between (A) regional [^18^F]flortaucipir BP_ND_, and MEG spectral measures and (B) regional [^11^C]UCB-JBP_ND_, and MEG spectral measures, in the frontal lobe (Spearman correlation coefficients). Colours represent different AD subjects, multiple frontal regions per subject are shown. Triangles represent right-hemisphere regions, circles left-hemisphere regions. Upon correcting for dependency of multiple ROIs per subject using GEE analyses, all correlations remained significant.

* *p* < 0.05; ** *p* < 0.01

**Supplementary Figure 4. Correlations between MMSE and averages of [^18^F]flortaucipir BP_ND_, [^11^C]UCB-J BP_ND_, MEG occipital absolute broadband power and MEG occipital relative alpha power.**


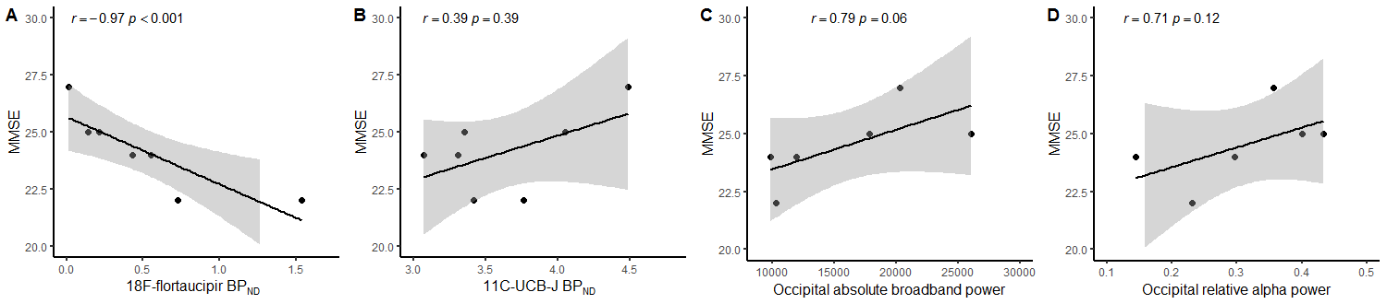


Shown are scatterplots of the correlations between MMSE and (A) volume-weighted average of [^18^F]flortaucipir BP_ND_,, (B) volume-weighted average of [^11^C]UCB-J BP_ND_, (C) average occipital absolute broadband power, and (D) average occipital relative alpha power (Spearman correlation coefficients and corresponding p-values).
